# Supplementary material for: Are carbon emissions trading and green financial instruments synergistic? -Comprehensive quantitative research based on content analysis
Source: PLoS One. 2024 Mar 7;19(3):e0298601. doi: 10.1371/journal.pone.0298601 (PMC10919601; doi:10.1371/journal.pone.0298601)
Supplement: S1 Data — (ZIP) [file pone.0298601.s001.zip › Data/地级市Z_F工作报告.zip!/║╙▒▒/└╚╖╗╩╨/└┤╫╘╣1⁄2╓┌║┼í╛┬φ┐╦╩2╛▌═°í┐_2011.docx]

各位代表：

现在，我代表廊坊市人民政府向大会作工作报告，请予审议，并请市政协委员和列席会议的同志提出意见。

一、“十一五”时期经济社会发展回顾

“十一五”时期，是我们面对诸多挑战、经受严峻考验并取得新胜利、实现新跨越的五年。全市人民在中共廊坊市委的正确领导下，以科学发展观统领经济社会发展全局，锐意进取，团结实干，“十一五”规划目标如期实现，一系列重大发展思路加速转化为现实成果，全市呈现出经济蓬勃发展、社会和谐稳定、人民安居乐业的可喜局面。胡锦涛总书记亲临廊坊视察并对我市工作给予充分肯定。

——过去的五年，是廊坊经济实力跃升新台阶，向强市行列大步迈进的五年。全市地区生产总值达到1331亿元，人均生产总值3.1万元，均实现了翻一番；全部财政收入从2005年的53.7亿元增加到195.4亿元，地方一般预算收入从24.3亿元增加到105.9亿元；规模以上工业增加值、全社会固定资产投资、城镇固定资产投资均实现翻两番，分别达到451.5亿元、909亿元、856.1亿元；社会消费品零售总额达到413.6亿元，增长2.3倍。五年中，全市经济总量由全省第七位上升到第六位，全部财政收入由第九位提升到第六位，地方一般预算收入由第七位跃居第四位；贷款余额由第六位提高到第三位，实现了从“小廊坊”到实力廊坊的历史性跨越。

——过去的五年，是廊坊经济全面提速，步入发展快车道的五年。我们积极应对金融危机等诸多困难，创造性地运用国家适度宽松货币政策、北京经济辐射能力增强、城镇面貌三年大变样三大机遇，有效破解了资金、产业、城市空间等难题，吸引了社会资金大量跟进。廊坊发展明显快起来、热起来了，实现了逆势崛起、弯道超越。全市实施亿元以上项目1372项，是“十五”期间的6.5倍。富士康、华为、润泽信息港等一批投资规模大、带动能力强、科技含量高的产业龙头项目落地建设，大连万达、香港新世界等众多大公司、大集团接踵而至。京津廊规划、产业、交通、通讯全方位对接，京廊同城发展迈出实质性步伐，全市经济形成了大发展快发展的强劲态势。

——过去的五年，是廊坊经济加速转型，产业结构发生巨大变化的五年。电子信息、装备制造、新能源等先进制造业规模不断壮大，电子信息产业增加值达到98.4亿元，占地区生产总值的比重达到7.4%，成为发展最快的新兴支柱产业。会展旅游、休闲商务、金融服务、健康医疗等现代服务业发展迅猛，服务业增加值达到461.5亿元，是2005年的2.5倍。科技创新能力明显提高，高新技术产业增加值193.5亿元，是2005年的2.8倍。农业产业化经营率达到64%。二氧化硫、化学需氧量完成目标任务，单位生产总值能耗、人均化学需氧量排放保持领先水平。具有鲜明廊坊特色的现代产业体系已具雏形。

——过去的五年，是廊坊城市功能显著增强，城乡建设全面突破的五年。我们大力推进城镇面貌三年大变样，实现了规划水准、环境质量、承载功能、风貌特色的整体提升，城镇化率达到48.8%。廊坊生态智能城市规划，荣获世界建筑界最高奖项——美国建筑师协会优异奖。市区拆临拆违基本完成，实施改造了20个城中村、8个棚户区、52个旧住宅小区，累计拆除650万平方米，占既有建筑面积的24%。市政基础设施投资完成227亿元，实施了156项重点城建项目，谋划建设了万达广场、广阳CBD等5个城市综合体项目，改造了28条城区道路，整治了34条背街小巷，打通了5条断头路。高标准建设了330个省级新民居示范村，启动实施了47个新民居组团，农村居民生产方式、生活环境、文明程度得到巨大改善和提升。我市获得了全国绿化模范城市、国家园林城市、国家环保模范城市和国家节水型城市等一批国家级荣誉。

——过去的五年，是廊坊环境优势更加彰显，基础支撑全面加强的五年。我们坚持打基础、造环境、蓄后劲，发展活力和竞争力持续增强。园区承载力显著提升，廊坊开发区升级为国家级开发区，燕郊开发区被批准为国家级高新区，新兴产业示范区、工业聚集区建设加快推进，省级以上园区数量和面积均居全省第一。交通建设投资完成113亿元，新增公路通车里程5824公里，分别是“十五”期间的7.1倍和2.8倍，9条高速公路直通京津，8个县（市、区）开通北京城市公交。南水北调配套工程加快推进，完成投资22亿元。市区绿化覆盖率达到46.5%，人均公共绿地面积12.7平方米，空气质量二级以上天数达到344天。信用体系建设迈出坚实步伐，荣获中国最佳信用环境城市奖。

——过去的五年，是人民生活持续改善，和谐社会建设开创新局面的五年。城镇居民人均可支配收入由10152元提高到20268元，农民人均纯收入由4621元提高到7589元，分别居全省第一位和第二位。城乡居民储蓄存款余额达到1168亿元，是2005年的1.7倍，城镇居民百户拥有汽车22.7辆，是2005年的5.7倍，城镇人均居住面积由25.3平方米增加到30平方米。持续实施“医、学、路、水、保、养”六项民生工程，五保户集中供养、城乡低保、孤儿救助等工作进入全国先进行列，城乡基本养老和医疗保险覆盖面不断扩大，新型农村合作医疗实现县乡村全覆盖。为1.1万户城市低收入家庭提供了住房保障，住房公积金覆盖率达到87.1%。在全省率先基本普及高中阶段教育和学前三年教育，全面实现了城乡免费义务教育。在全省率先完成农村卫生“三基两化”建设任务，抗击“甲流”取得重大胜利，城乡公共文化服务体系建设成果丰硕。成功举办第十三届省运会并创历史最好成绩，“大气、锐气、和气”的廊坊精神得到充分展现。安全生产主要指标连续五年全省领先，食品药品安全、社会稳定工作得到加强，圆满完成北京奥运、国庆六十周年等重大活动安保任务。统计、审计、工商、物价、人防、气象、人口、民族宗教、妇女儿童、外事侨务、广播电视、防震减灾等各项工作均取得了新成绩。

2010年是“十一五”的最后一年。全市上下紧紧抓住难得的发展机遇，冲刺跨越，全面争先，各项工作再创佳绩。全市地区生产总值增长12.5%；全部财政收入和地方一般预算收入分别增长36.3%和52.8%，增速均为全省第一；城镇居民人均可支配收入和农民人均纯收入分别增长10.6%和11%。

各位代表，“十一五”的发展，为全面建设小康社会奠定了坚实基础，谱写了廊坊改革开放和现代化建设的辉煌篇章。这些成就的取得，是中共廊坊市委统揽全局、正确领导的结果，是人大、政协和社会各界大力支持、全市人民拼搏奋斗的结果。在这里，我代表廊坊市人民政府，向人大代表、政协委员、各民主党派、工商联及全市人民，向驻廊中省直单位、人民解放军、武警官兵和政法干警，向关心廊坊、支持廊坊的中外客商和朋友们，表示衷心的感谢！

“十一五”时期的工作实践，让我们深刻认识到，推进全市经济社会又好又快发展，实现富民强市的宏伟目标，必须把加速发展、跨越争先作为根本任务；必须把转变发展方式、调整产业结构作为工作主线；必须把增强核心竞争力和可持续发展能力作为重要支撑；必须把深化改革和扩大开放作为强大动力；必须把保障和改善民生作为根本出发点和落脚点。在今后工作中，只要我们坚定不移地走科学发展的道路，不断在实践中坚持和发扬好的经验和做法，就一定能在未来发展中迈出更大步伐，取得更加丰硕的成果！

二、“十二五”时期思路目标和主要任务

“十二五”时期，是全面建设小康社会的关键时期。新的五年，新的征程。廊坊处于大有作为的战略机遇期。国际产业结构调整和国内“南资北移”明显加速，京津冀一体化进程不断加快，北京疏解城市压力、拓展发展空间的需求日益增强，环首都经济圈战略全面实施，廊坊独特的区位优势正在加快转化为发展优势。廊坊处于率先发展的重要跨越期。经过“十一五”的持续发展和积累，我市现代产业、城镇和交通体系已具雏形，新的经济增长点加速形成，发展基础前所未有，发展后劲空前强大，标志着我市已经进入跨越性发展的新阶段。廊坊处于经济转型的全面攻坚期。在国家宏观政策趋向收紧、区域竞争压力增大的形势下，我们面临着加快转变经济发展方式这一艰巨的任务，需要我们树立强烈的忧患意识，充分估计各种困难和不利因素，着力解决产业体系不完善、传统产业技术水平低、资源能源约束加剧等突出问题，以攻坚克难的锐气、战无不胜的信念，努力在京津冀城市群抢占先机、率先发展。
